# High Long-term Survivorship From Revision After Physeal-Sparing “Over-the-Top” Anterior Cruciate Ligament Reconstruction and Lateral Tenodesis in Skeletally Immature Patients: 8- to 17-Year Follow-up

**DOI:** 10.1177/23259671251389126

**Published:** 2025-12-02

**Authors:** Alberto Grassi, Giacomo Dal Fabbro, Stefano Di Paolo, Francesco Aparo, Mohammad Ibrahim Allhalalmeh, Gian Andrea Lucidi, Stefano Zaffagnini

**Affiliations:** * IRCCS Istituto Ortopedico Rizzoli, 2nd Orthopaedic Department, Bologna, Italy; † Dipartimento di Scienze Biomediche e Neuromotorie DIBINEM, Bologna University, Italy; Investigation performed at Rizzoli Orthopaedic Institute, Bologna, Italy

**Keywords:** ACL, skeletally immature, skeletal age, physeal sparing, over the top, LET, long term, failure, reoperation, PROs

## Abstract

**Background:**

While the rate of anterior cruciate ligament (ACL) injuries and reconstruction procedures in children and adolescents is increasing, evidence of long-term outcomes after ACL reconstruction in skeletally immature patients is still lacking.

**Purpose:**

To assess the long-term survivorship from revision and reoperations and to analyze the functional results and patient-reported outcomes (PROs) in skeletally immature patients (with an open physis present at magnetic resonance imaging evaluation) who underwent ACL reconstruction with hamstrings tendon with an over-the-top (OTT) technique and a lateral extra-articular tenodesis (LET).

**Study Design:**

Case series; Level of evidence, 4.

**Methods:**

The database of a single institution was retrospectively searched for skeletally immature patients who underwent ACL reconstruction. Ipsilateral and/or contralateral reoperations were recorded. Knee injury and Osteoarthritis Outcome Score (KOOS), Lysholm, and Tegner Activity Level scores were collected at final follow-up. Survivorship was inspected through Kaplan-Maier method with ipsilateral ACL revision as endpoint. Differences in demographics and PROs were assessed through Student *t* test.

**Results:**

A total of 43 patients (mean age at surgery, 13.3 ± 1.6 years), all of whom underwent an OTT technique associated with a LET, at mean follow-up of 11.0 ± 2.7 years were included. Four patients (9%) underwent revision ACL in the ipsilateral knee at a mean of 5.3 ± 2.4 years after surgery, with a revision rate of 5% at 5-year and 10% at 10- and 15-year follow-ups. Four patients (9%) underwent arthroscopy for a new meniscal tear, and a further 5 patients (12%) underwent staple removal because of local discomfort. A total of 11 patients (26%) underwent ≥1 reoperation in the ipsilateral knee after a mean of 3.0 ± 2.1 years; and 8 patients (19%) underwent contralateral ACL reconstruction after a mean of 3.7 ± 3.0 years after surgery. Mean KOOS subscales were all above the Patient Acceptable Symptom State. Patients <13 years old at surgery showed worse Lysholm (82.0 vs 94.6; *P* = .025) and KOOS–Activities of Daily Living (96.7 vs 99.9; *P* = .025) compared with those ≥13 years.

**Conclusion:**

Patients showed high survivorship (90% of cases) from ACL revision at long-term follow-up. Still, more than one-quarter (26%) of skeletally immature patients who underwent physeal-sparing OTT plus LET technique needed a further operation in the ipsilateral knee. Higher rate of hardware removal procedures and lower functional reported outcomes were detected in patients aged <13.

In the past 20 years, the incidence of anterior cruciate ligament (ACL) injuries among skeletally immature populations has increased,^22^ along with the efforts in defining the best treatment approach for these kinds of patients.^15,18^ The significant risk of new meniscal and chondral tears together with poor reported outcomes after nonoperative or delayed surgical protocol^16,23^ has resulted in an increase of the incidence of reconstruction procedures among children and adolescents who sustained ACL rupture.^4^ However, the increased risk of osteoarthritis development at long-term follow-up^10^ and damage to the open physis due to tunnel drilling remain major concerns of surgeons performing ACL reconstruction in children and adolescents, and various techniques have been proposed to decrease the risk of growth disturbance after ACL reconstruction in skeletally immature patients.^1,27^ The over-the-top (OTT) femoral fixation technique has been shown to control anterior and rotatory laxity without the need to drill a femoral tunnel.^14,19^ Promising results have been shown in skeletally immature patients who underwent ACL reconstruction with an OTT technique at short- and midterm follow-up,^25^ but evidence of long-term survivorship and outcomes after OTT physeal-sparing procedures is still lacking.

The higher risk of failure in younger compared with older patients represents another factor that should be considered in the setting of pediatric ACL reconstruction.^12,30^ While the addition of a lateral extra-articular tenodesis (LET) has been shown to be effective in reducing the risk of rerupture in pivoting and high-demanding skeletally mature patients,^8^ the role of LET in skeletally immature patients and its effectiveness at long-term follow-up has not been well studied.^3,7^

Thus, the aim of the present study was to assess the survivorship from ACL rerupture of physeal-sparing OTT ACL reconstruction combined with a LET in skeletally immature patients at long-term follow-up. Furthermore, the rate of ipsilateral reoperation, contralateral ACL rupture, and patient-reported outcomes (PROs) were investigated at final follow-up. The hypothesis was that skeletally immature patients who underwent physeal-sparing ACL reconstruction with an OTT technique would have a high survivorship from reinjury and reoperation, with good PROs at final follow-up.

## Methods

The study protocol was approved by the institutional review board of the authors’ institution. All parents of the underage patients (<18 years) signed the informed consent form.

The electronic patient database of the authors’ institution was searched for skeletally immature patients undergoing ACL reconstruction between 2006 and 2016. Patients were considered skeletally immature if they had open femoral and/or tibial physes seen on preoperative magnetic resonance imaging (MRI). Tanner stage was not routinely assessed. Additional inclusion criteria were clinical and radiographic evidence for ACL rupture; regular participation in sport activities; asymptomatic, stable, and functional contralateral knee; body mass index <30 kg/m^2^; minimum follow-up of 8 years, which we considered as minimum follow-up that can be defined as long-term follow-up. The exclusion criteria were local or systemic infection, knee osteoarthritis documented on preoperative imaging or intraoperatively, neuromuscular disorders, or anaphylactic reactions. Some of the patients included in the current study were analyzed in a previous study at shorter follow-up.^25^

Demographic data of the patients and meniscal status at the time of the index surgery were recorded. Patient characteristics and details were obtained by chart review and all included patients were contacted by phone. The PROs were obtained through an online survey delivered by email.

### Surgical Technique

**Figure 1.**
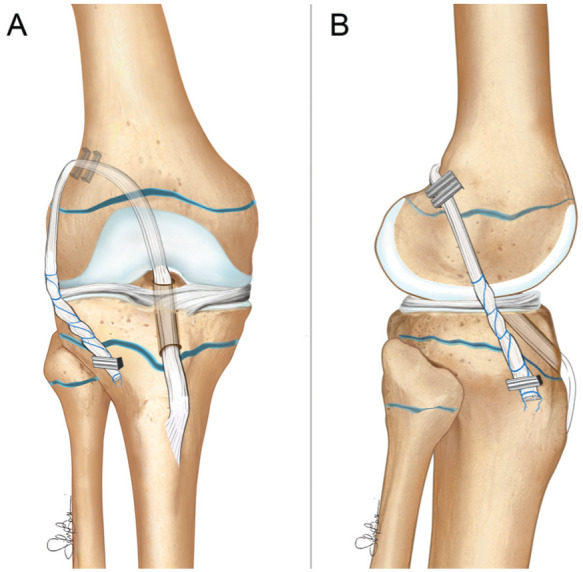
Physeal-sparing over-the-top anterior cruciate ligament reconstruction with hamstring tendon autograft and lateral tenodesis. (A) Anteroposterior view and (B) lateral view.

All patients underwent ACL reconstruction with a physeal-sparing OTT plus lateral tenodesis technique using hamstring tendons previously described in the literature^25^ (Figure 1). It represents a modification of a similar technique previously described by Zarins and Rowe.^32^ Intraoperative fluoroscopy was used to check the position of the tibial tunnel and of the staple used to secure the LET, which were just above and below the tibial growth plate, respectively. This surgical procedure still represents the routine treatment for ACL rupture in skeletally immature patients in the authors’ clinical practice. All surgeries were performed by the senior author (S.Z.).

### Assessed Outcomes

Ipsilateral reoperations, ACL reconstruction failures (defined as ipsilateral revision ACL), hardware removal procedures, and contralateral ACL ruptures were investigated. Visual analog scale for pain at rest and during activity, Lysholm score, Tegner Activity Rating Scale, and the Knee injury and Osteoarthritis Outcome Score (KOOS) with its subscales were collected at final follow-up. Patients who underwent ACL revision were included in the analysis. The KOOS score subscales were compared with the Patient Acceptable Symptom State (PASS) at the 10-year follow-up recently reported in the literature,^29^ which have been defined as 59, 76.5, 93.8, 71.6, and 85.4 for Quality of Life, Symptoms, Activities of Daily Living, Sport and Recreation, and Pain, respectively.

### Rehabilitation Protocol

The rehabilitation protocol consisted of 1 month with progressive weightbearing on the operated limb with the aid of 2 crutches, followed by 2 additional weeks with the use of 1 crutch contralateral to the operated side. Physical therapy–assisted exercise for joint range of motion recovery was initiated in the early postoperative days (about 3-4 days) in the absence of associated meniscal lesions. On the other hand, in cases of meniscal repair, for the first postoperative month, patients were advised to use reduced loading (from 10% to 50% depending on the intraoperatively detected meniscal status) on the treated knee and to wear a brace with range of motion allowed from 0° to 90° for the first 2 weeks after surgery. After complete wound healing (15-18 days), patients could begin functional recovery with hydrotherapy and low-impact activities, with progressive improvement of knee flexion-extension mechanism. Patients were generally allowed to return to sports 10 months after surgery in high-impact sports activities, such as football or basketball.

### Statistical Analysis

The statistical analysis was performed using the RStudio (Version 4.3.2; Posit PBC). The normal distribution of data was inspected through the Shapiro-Wilk test. Normally distributed continuous variables are presented through mean and standard deviation, while nonnormally distributed and categorical variables are presented as median and interquartile range or percentage over the total. Survivorship was inspected through the Kaplan-Maier method. Differences in demographics and PROs were assessed through the Student *t* test, while the Friedman test was adopted to inspect the difference in Tegner activity level score among preinjury, presurgery, postsurgery, and final follow-up. Conover test with Bonferroni correction for post hoc comparison was used to assess differences between single time points. The significance level was set as a *P* value < .05.

## Results

### Patient Characteristics

**Figure 2.**
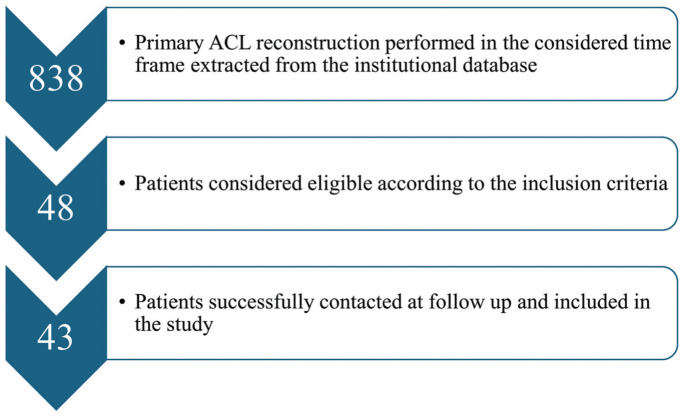
STROBE (Strengthening the Reporting of Observational Studies in Epidemiology) flow diagram. ACL, anterior cruciate ligament.

On the whole, 838 primary ACL reconstructions were performed in the considered time frame. After the application of inclusion and exclusion criteria, 48 patients were considered eligible for this study. Five patients were lost to follow-up; thus, the overall follow-up rate was 90% (Figure 2).

A majority of patients were male (74%) and the mean age at surgery was 13.3 ± 1.6 years (range, 8-15). The mean follow-up at final evaluation was 11.0 ± 2.7 years, and thus the mean age of patients at this time point was 24.3 ± 1.9 years.

ACL reconstruction was performed using the same technique in all patients (100%); 9 of the total 43 (21%) had medial meniscal tear while 13 (30%) had lateral meniscal tear (Table 1).

**Table 1:** Demographics and Surgical Characteristics of Patients Included in Study*^*a*^*

Age at surgery, y	13.3 ± 1.6
<13	10 (23)
≥13	33 (77)
Age at final follow-up, y	24.3 ± 1.9
Follow-up, y	11.0 ± 2.7
Sex, M/F	32 (74)/11 (26)
Medial meniscal injury	9 (21)
Repair	7
Meniscectomy	2
Lateral meniscal injury	13 (30)
Repair	6
Meniscectomy	7

a Data are presented as mean ± SD, n, or n (%). F, female; M, male.

### Ipsilateral Reoperations

**Figure 3.**
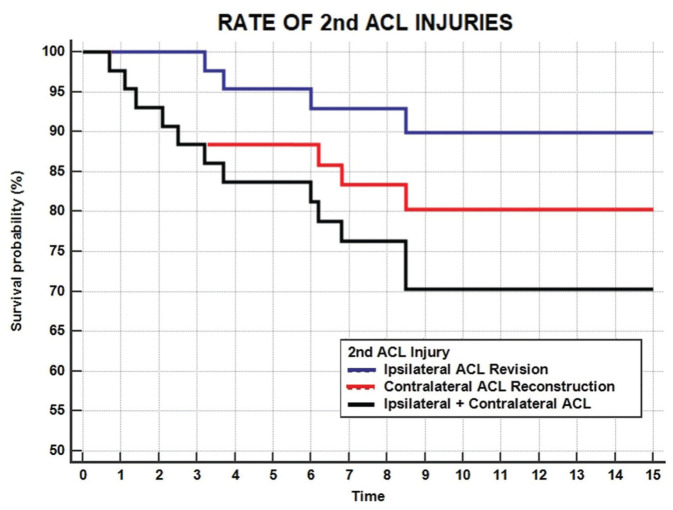
Rate of ipsilateral ACL revision (blue dotted line), contralateral ACL reconstruction (red dotted line), and overall second ACL injury (black line). ACL, anterior cruciate ligament.

A total of 4 patients (9%) underwent revision ACL reconstruction in the ipsilateral knee at a mean of 5.3 ± 2.4 years after surgery. Survival rates of ACL reconstruction were 100% at 1 year, 100% at 2 years, 95% at 5 years, 90% at 10 years, and 90% at 15 years (Figure 3, Table 2).

**Table 2:** Survival Rates for Ipsilateral, Contralateral, or Total ACL Second Injuries at Various Follow-ups*^a^*

	1 Year	2 Years	5 Years	10 Years	15 Years
Ipsilateral ACL revision	100	100	95	90	90
Contralateral ACL reconstruction	98	93	88	80	80
Second ACL injury	98	93	84	70	70
Male^*b*^	97	91	78	55	55
Female^*b*^	100	100	100	82	82

a Data are presented as percentages.

b The percentage free of any injury.

Another 4 patients (9%) underwent knee arthroscopy for a new meniscal tear at a mean of 2.2 ± 0.8 years after the index surgery: 2 medial meniscectomies after failed medial meniscal repair, 1 lateral meniscal rerepair after failed lateral meniscal repair and 1 partial lateral meniscectomy after a new lateral meniscal tear.

A further 5 patients (12%) underwent staple removal because of local discomfort at a mean time of 2.2 ± 1.0 years after surgery. Patients aged <13 years at surgery had a higher rate of staple removal (40%) with respect to those aged ≥13 years (3%) (*P* = .0008) (Figure 5).

**Figure 4.**
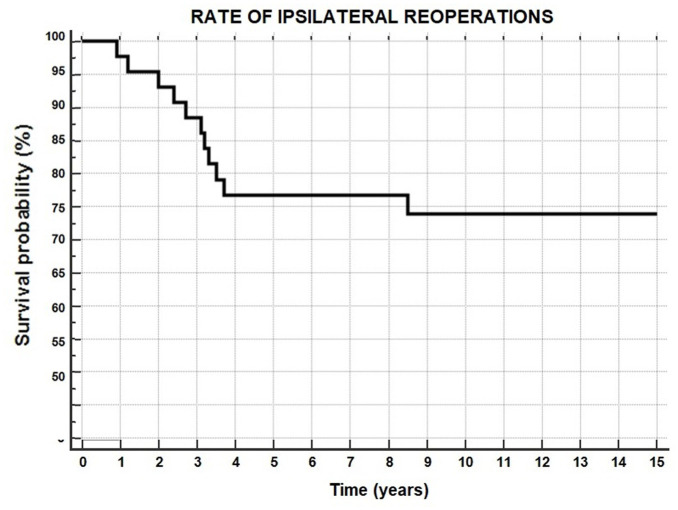
Rate of reoperation in the ipsilateral knee.

Considering the latter surgeries, a total of 11 patients (26%) underwent ≥1 reoperation in the ipsilateral knee during the considered follow-up, after a mean of 3.0 ± 2.1 years. Two patients had >1 reoperation: 1 patient underwent staple removal and subsequent ACL revision associated with lateral meniscal repair, and in the other patient a subsequent lateral meniscectomy was performed first, followed by staple removal. The overall survival from reoperation was 98% at 1 years, 95% at 2 years, 77% at 5 years, 74% at 10 years, and 74% at 15 years (Figure 4, Table 2).

**Figure 5.**
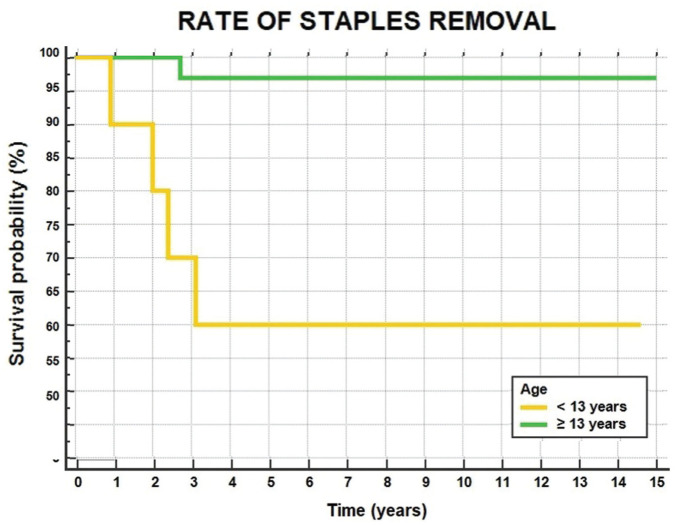
Rate of staple removal in patients <13 years of age (yellow line) or ≥13 years (green line).

### Second ACL Injury

A total of 8 patients (19%) underwent contralateral ACL reconstruction after a mean of 3.7 ± 3.0 years after surgery. The survival from contralateral ACL reconstruction was 98% at 1 year, 93% at 2 years, 88% at 5 years, 80% at 10 years, and 80% at 15 years (Figure 3, Table 2).

**Figure 6.**
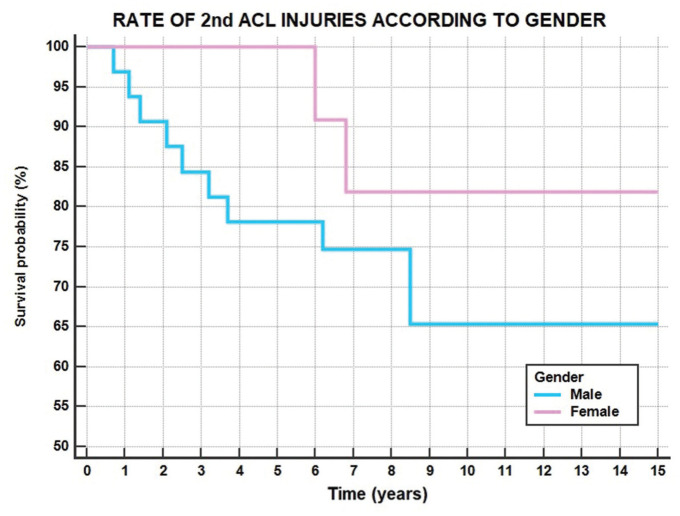
Rate of second ACL injuries in male (blue line) and female (pink line) patients. ACL, anterior cruciate ligament.

Considering also the previously reported 4 ipsilateral ACL revisions, a total of 12 patients (28%) experienced a second ACL injury after a mean of 4.2 ± 2.8 years from surgery. The overall survivorship from second ACL injury was 98% at 1 year, 93% at 2 years, 84% at 5 years, 70% at 10 years, and 70% at 15 years (Figure 6, Table 2).

### Patient-Reported Outcomes

Mean PROs were higher than the PASS in all KOOS subscales (Table 3). Specifically, 43 patients (100%) achieved PASS in the Quality of Life subscale, 40 (92.6%) in the Symptoms subscale, 39 (90.7%) in the ADL subscale, 41 (97.2%) in the Sport and Recreation subscale, and 40 (97.8%) in the Pain subscale.

**Table 3:** Patient-Reported Outcomes According to Sex and Age*^*a*^*

	Overall	Male (n = 32)	Female (n = 11)	*P*	Age <13 (n = 33)	Age ≥13 (n = 10)	*P*
VAS							
At rest	0.8 ± 2.2	0.9 ± 2.4	0.5 ± 1.8	.63	1.5 ± 3.2	0.6 ± 1.8	.28
With activity	1.3 ± 2.3	1.2 ± 2.4	1.5 ± 2.3	.76	1.6 ± 3.2	1.2 ± 2.0	.61
Lysholm	91.5 ± 15.7	91.7 ± 16	91.1 ± 15.5	.92	82.0 ± 23.4	94.6 ± 11.1	**.025**
KOOS							
QOL	93.1 ± 12.6	93.9 ± 12.2	91.1 ± 14.1	.53	92.1 ± 14.4	93.5 ± 12.2	.77
Symptoms	92.6 ± 16.3	92.6 ± 17.3	92.4 ± 13.9	.97	85.3 ± 22.4	94.9 ± 13.4	.10
ADL	99.1 ± 4.0	99.1 ± 4.6	99.2 ± 2.2	.90	96.7 ± 8.0	99.9 ± 0.3	**.025**
Sport/Rec	97.2 ± 7.8	97.2 ± 7.9	97.2 ± 7.8	.99	96.9 ± 8.2	97.3 ± 7.8	.88
Pain	97.8 ± 5.4	97.8 ± 5.8	97.8 ± 4.4	.99	95.9 ± 8.4	98.4 ± 4.1	.22

a Bold *P* values represents statistically significant differences between the groups. ADL, Activities of Daily Living; QOL, Quality of Life; Sport/Rec, Sport and Recreation.

No differences in PROs emerged between male and female patients (*P* > .05). Patients aged <13 years at surgery reported significantly decreased Lysholm (82.0 vs 94.6; *P* = .025) and KOOS ADL (96.7 vs 99.9; *P* = .025) compared with those aged ≥13 years (Table 3).

**Figure 7.**
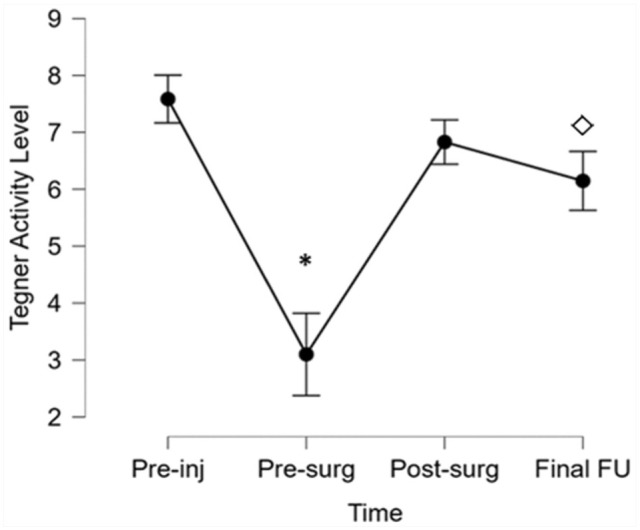
Tegner activity level score changes over time. Asterisk represents differences between presurgery (Pre-surg) time and all other time points (*P* < .001); diamond represents differences between preinjury (Pre-inj) level and final follow-up (FU). Post-surg, postsurgery.

Median Tegner activity level score at final follow-up (6.2 [0-9]) was significantly higher than before surgery (3.2 [1-9]; *P* < .001) but lower than the preinjury level (7.6 [2-9]; *P* = .005) (Figure 7).

Considering the 4 patients who underwent ipsilateral revision ACL and the 3 patients who were scored as “poor” according to the Lysholm score, the remaining 36 out of 43 patients (84%) were considered to have a successful long-term outcome of physeal-sparing ACL reconstruction.

## Discussion

The main finding of the current study is that physeal-sparing OTT ACL reconstruction with LET in skeletally immature patients provided a survivorship free from ipsilateral revision ACL reconstruction of 90% at 10 and 15 years of follow-up. The ipsilateral reoperation rate remained high, with the main cause of reoperation being staple removal. Including the contralateral side, 28% of the patients had a second ACL injury after a mean of 4 years from the first surgery. Younger patients were associated with a greater risk of need for a hardware removal procedure and lower PROs.

The rate of ipsilateral ACL reinjury at long-term follow-up found in the present study is lower than previously reported in the literature after physeal-sparing ACL reconstruction using hamstring tendon autograft. A prospective case series of 12 patients with a minimum 15-year follow-up showed a failure rate of 25% after an OTT femoral fixation procedure, with all the rerupture episodes after growth plate closure due to a new sports trauma.^7^ Calvo et al^3^ performed a retrospective analysis among 27 patients at a mean 11 years of follow-up after a transepiphyseal ACL reconstruction with vertically oriented tibial and femoral tunnels, recording a revision ACL rate of about 14%. Conversely, a survivorship similar to the present study was reported at midterm follow-up by Bonnard et al^2^ after a periosteum–patellar tendon–periosteum autograft reconstruction according to the Clocheville technique in a retrospective cohort series of 57 patients. However, while the authors reported ipsilateral recurrent ACL injury in 5.4% at mean of 5.5 years, they did not provide a long-term follow-up analysis. The relatively small sample size of the current study might be responsible for the failure to detect significant role of potential risk factors among patient demographics and level of activity. The clear role of sex, level of sports, and rehabilitation protocol in affecting the ACL reconstruction failure rate still need to be investigated and cleared in further studies with stronger design and larger sample size.

Unlike previous long-term studies using hamstring tendons for physeal-sparing ACL reconstruction,^3,7^ in the present study a LET was performed in association with the intra-articular reconstruction. Reasons why the addition of a LET procedure in skeletally immature patients is worth considering have been recently highlighted in the literature. An MRI-based cohort study comparing all-epiphyseal ACL reconstruction with quadriceps tendon autograft in skeletally immature athletes revealed an improved graft maturity at 2 years postoperatively in patient with associated LET compared with isolated ACL reconstruction after controlling for both surgical technique and sex.^24^ Furthermore, in a multicentric cohort study including 66 pediatric patients who underwent ACL reconstruction with hamstring graft, the addition of a modified Lemaire LET significantly reduced the cumulative failure rate at 2-year follow-up compared with patients who underwent isolated intra-articular procedure.^21^ Moreover, a cohort study including 111 adolescent patients showed a lower graft rupture rate after hamstring ACL reconstruction with LET compared with an isolated intra-articular reconstruction at a mean 3 years of follow-up.^17^ The present study further confirms these promising short-term outcomes, showing a higher survivorship even at very long-term follow-up in patients with an associated LET compared with previous studies in which an isolated intra-articular ACL reconstruction with hamstring was performed.^3,7^

The overall ipsilateral reoperation rate was 26%. These data are similar to what was recently recorded with a shorter follow-up among a case series of 85 skeletally immature patients after a quadriceps tendon ACL reconstruction, reporting subsequent surgical procedures in 24%.^6^ In the present study, about half of the reoperations consisted of a staple removal procedure. Furthermore, it is worth mentioning that the need for hardware removal was up to 40% among patients <13 years old, with a significant difference compared with older patients. The actual reasons for this difference cannot be derived from the data in the present study. However, in our opinion, the increased proximal staple migration due to the greater bone growth after surgery in younger patients, associated with the larger size of the staple relative to the bone and a smaller muscle mass, may have been responsible for the higher rate of hardware removal procedures. This result aligns with the trend found by Cordasco et al^5^ in a previous prospective analysis where prepubescent athletes showed a higher rate of reoperation excluding revision ACL reconstruction than young adolescents at a ≥2-year follow-up after all-epiphyseal or partial transphyseal hamstring reconstruction. However, Cordasco et al^5^ did not find this difference to be statistically significant, and they cited a new meniscal lesion as the main reason for reoperation rather than the need for hardware removal. It should be taken into account that failure of meniscal repair was also a common cause of reoperation in the present study, with 3 out of 13 patients undergoing an associated meniscal repair needing subsequent surgery because of meniscal repair failure. Therefore, it is important to adequately inform the skeletally immature patient before performing a physeal-sparing OTT technique with LET, knowing the high risk of a subsequent procedure if <13 years old, because of the need for hardware removal or failure of meniscal repair. Furthermore, the use of alternative fixation devices should be considered in the future to reduce the rate of subsequent hardware removal procedures.

The association between a younger age and an increased risk of graft failure and contralateral ACL injury after primary ACL reconstruction has been widely described.^8,9,11,12,31^ In the present study, including also the contralateral knee, 28% of the patients underwent a second ACL injury after a mean of about 4 years of follow-up. This rate compares favorably with results previously reported in the literature by shorter follow-up analysis.^5,26^ A cohort study by Sasaki et al^26^ including 102 skeletally immature patients revealed a second ACL injury rate between 28% and 38% at 3 years of follow-up after both all-epiphyseal and standard double-bundle hamstring tendon ACL reconstruction. Thus, the current data, according to the outcomes reported in the literature, pointed out the high rate of a second ACL injury at long-term follow-up after primary reconstruction also in skeletally immature patients and confirmed that the risk of a second ACL injury should be always considered in the setting of ACL reconstruction in pediatric and young adolescent athletes.

Physeal-sparing OTT ACL reconstruction with LET was associated with excellent PROs at long-term follow-up. These results confirm the previously published findings of the same technique at midterm follow-up^13,25^ and show the long-lasting effect of this procedure on symptoms and knee function. These findings are similar to those previously reported in the literature for both short- and long-term follow-up. Nikolau et al^20^ found a mean Lysholm score of 89 among 94 skeletally immature patients at 3 years of follow-up, while Calvo et al^3^ reported a mean Lysholm score of 94 at 10 years of follow-up among their case series. Interestingly, in the present study, significantly lower Lysholm and KOOS ADL subscale score were recorded in patients <13 years old at the time of surgery. Conversely, Cordasco et al^5^ found a lower return-to-sport rate at short-term follow-up in young adolescents than in prepubescent athletes. It should also be noted that in the current study, there was a tendency for the Tegner score to decline, and it was not possible to determine whether this was primarily related to the age of the patients or whether it was due to poorer knee function. Therefore, while the findings of the present study suggest that an overall lower subjective knee function can be expected in younger skeletally immature patients undergoing OTT ACL reconstruction with LET, the relationship between age and functional outcomes in the setting of skeletally immature patients undergoing ACL reconstruction remains controversial. Similarly, clear evidence about the difference between clinical outcomes and safety in terms of growth disturbance and alignment deformity after an all-epiphyseal technique compared with a standard transphyseal technique is still lacking.^26^

### Strengths and Limitations

The main strength of this study is the long-term follow-up analysis of the same surgical procedure performed by the same high-volume senior knee surgeon, along with the relatively large cohort compared with the available literature. Nevertheless, this study is limited by its retrospective design, which makes it susceptible to selection and detection bias, and by the lack of objective physical evaluation data and routine graft imaging. Data collection was individually performed by the investigators utilizing internal database coding; therefore, despite the cross-referenced check, some patients may have been missed due to incorrect coding, leading to a potential selection bias. Moreover, despite the KOOS scale's having been defined as effective and sensitive in measuring improvements after intervention, the Pain subscale is characterized by a higher “ceiling effect” that decreases its validity.^28^ The nonuse of a specific score for pediatric populations, such as the Pediatric International Knee Documentation Committee, represents a flaw of the present study and should be considered for further analysis in this cohort of patients. Furthermore, a selection bias that could affect our study is that most enrolled participants were male patients. Moreover, another limitation of this study could be represented by the lack of randomization or a comparison group. The evolution in the rehabilitation approaches among the considered time frame should also be acknowledged. Last, radiographic evaluation detecting postoperative growth disturbance and deformity was not performed in the current case series. However, the safety of the physeal-sparing ACL reconstruction OTT and LET techniques performed in the present study with regard to growth disturbance and alignment deformity was previously reported.^25^

## Conclusion

The physeal-sparing OTT ACL reconstruction with LET provided skeletally immature patients with a high survivorship (90%) from graft failure at >8 years follow-up, while 26% needed ≥1 reoperation in the ipsilateral knee. A higher rate of hardware removal procedures and lower PROs can be expected in patients aged <13.
